# A Comparison Between Culprit Versus Complete Revascularization in Diabetic Patients With Acute Myocardial Infarction

**DOI:** 10.1002/clc.70046

**Published:** 2024-11-14

**Authors:** Naser Aslanabadi, Sina Mashayekhi, Maziar Rezvani, Ali Abdollahzadeh, Amirhossein Hajialigol

**Affiliations:** ^1^ Department of Cardiology, Cardiovascular Research Center Tabriz University of Medical Sciences Tabriz Iran; ^2^ School of Medicine Tabriz University of Medical sciences Tabriz Iran; ^3^ Alborz Office of Universal Scientific Education and Research Network (USERN) Alborz University of Medical Sciences Karaj Iran

**Keywords:** acute myocardial infarction, complete revascularization, diabetes mellitus, STEMI

## Abstract

**Introduction:**

The benefit of complete revascularization in diabetic patients with myocardial infarction remains unclear. this study aims to find the optimal strategy of total vascular repair for diabetic patients with acute myocardial infarction.

**Methods:**

In an analysis of a cohort, we assigned diabetic patients with myocardial infarction who were undergoing percutaneous coronary intervention (PCI) of the culprit lesion to receive either staged complete revascularization of nonculprit lesions or to receive no further revascularization in Madani Hospital (Tabriz, Iran). Functionally significant nonculprit lesions were identified either by angiography. The primary outcome was rates of readmission, cardiac deaths, nonfatal myocardial re‐infarction, and overall mortality at 1 year.

**Results:**

In our center, a total of 1186 patients underwent primary‐PCI treatment, among which 521 were diagnosed with diabetes. Ultimately, 393 patients were selected for inclusion in the study. Within this cohort, 271 individuals (68.9%) underwent repair of only the culprit vessels (group 1), while 122 individuals (31.1%) received a comprehensive staged restoration of the vessels (group 2). During this time, group 1 in comparison with group 2 experienced 204 (75.3%) versus 97 (79.5%) cases of readmission, 48 (17.7%) versus 8 (6.5%) instances of cardiac death, 22 (8.1%) versus 18 (14.7%) occurrences of nonfatal myocardial re‐infarction. Notably, the incidence of cardiac death in group 2 were significantly lower than that in group 1 (*p* ≤ 0.05).

**Conclusions:**

For individuals diagnosed with diabetes, staged complete revascularization demonstrated a lower frequency of readmission, cardiac deaths, nonfatal myocardial reinfarction, and overall mortality, in contrast to revascularization that targeted only the culprit lesion.

## Graphical abstract

In our center, 271 individuals underwent repair of only the culprit vessels, while 122 received a staged restoration. group 1 in comparison with group 2 experienced 75.3% versus 79.5% cases of readmission, 17.7% versus 6.5% instances of cardiac death, 8.1% versus 14.7% occurrences of nonfatal myocardial re‐infarction.

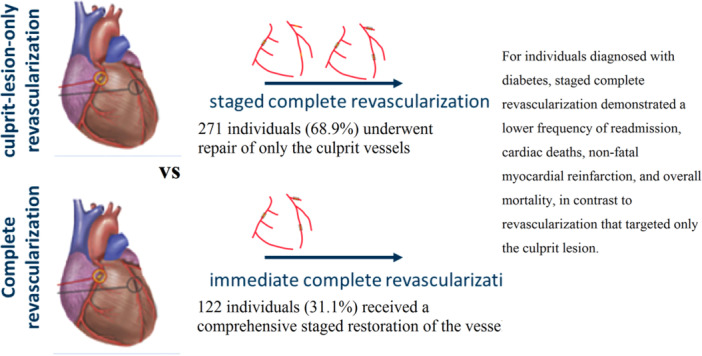

**Abbreviations**
AMI: acute myocardial infarction
CAD: coronary artery disease
CR: culprit‐only revascularization
LBBB: left bundle branch block
LVEF: left ventricular ejection fraction
MVD: multivessel disease
PCI: percutaneous coronary intervention
STEMI: ST‐segment elevation myocardial infarction
TIMI: thrombolysis in myocardial infarction

## 1 Introduction

Acute myocardial infarction (AMI), commonly referred to as a heart attack, is a critical medical condition characterized by the sudden interruption of blood flow to part of the myocardium, the etiology is usually due to the blockage of a coronary artery by a blood clot [1]. This interruption in blood flow can cause significant damage to the myocardium and lead to severe complications and death [2]. According to the World Health Organization, AMI is one of the leading causes of death worldwide and affects millions of people every year [3]. The prevalence of AMI is increasing in part due to increasing rates of risk factors such as hypertension, diabetes, and obesity [4]. timely and effective treatment of AMI is critical to minimize myocardial damage and improve patient survival outcomes. Immediate interventions such as percutaneous coronary intervention (PCI) or thrombolytic therapy have been shown to significantly reduce mortality and improve recovery in AMI patients [2, 5, 6]. On the other hand, delay in treatment is associated with worse outcomes and shows the importance of prompt diagnosis and prompt treatment measures in the management of AMI [7].

Diabetes is a known risk factor for cardiovascular diseases, including AMI [8]. The relationship between diabetes and cardiovascular diseases is complex and multifactorial and includes a combination of metabolic, inflammatory, and hemodynamic abnormalities that accelerate atherosclerosis and increase vulnerability to plaque rupture and thrombosis [2, 9, 10]. Studies have shown that the risk of cardiovascular disease in people with diabetes is two to four times higher than people without diabetes [10, 11].

In the context of AMI, diabetic patients not only present more often with multivessel disease (MVD), but also experience worse clinical outcomes [11, 12]. This includes higher rates of heart failure, reinfarction and mortality [12]. The hyperglycemic milieu in diabetic patients negatively affects endothelial function, promotes inflammation, and exacerbates myocardial injury, thereby complicating both acute management and long‐term prognosis of AMI [13]. Furthermore, diabetes impairs the efficacy of standard AMI treatments such as PCI and thrombolysis, leading to higher rates of side effects and complications during and after these procedures [14].

Consequently, diabetic patients with AMI require careful management and often require more aggressive and tailored treatment strategies to improve their outcomes [15]. Culprit‐only revascularization (CR) is a standard procedure in the treatment of AMI, especially in cases involving ST‐elevation myocardial infarction (STEMI) [2]. This strategy involves performing PCI to restore blood flow exclusively in the artery responsible for the infarction, known as the culprit artery [16]. The primary goal is to rapidly restore blood supply to the ischemic myocardium, thereby limiting myocardial damage and improving short‐term survival [17]. The advantages of this method include reducing the operation time, reducing the immediate risk of complications and faster recovery [18]. Additionally, focusing solely on the culprit artery can be beneficial in patients who are hemodynamically unstable and may not tolerate long‐term procedures. However, the limitation of this strategy is that it leaves any significant stenosis in nonculprit arteries untreated, which may predispose patients to future cardiovascular events [17, 18]. Studies have shown that patients with MVD, which is common among people with diabetes, often experience worse long‐term outcomes when only the culprit artery is treated [2, 16].

Complete revascularization, an emerging method in the management of AMI, involves performing PCI on all significantly narrowed coronary arteries, not just the culprit artery; this strategy aims to reduce the overall ischemic burden and potentially improve long‐term outcomes by addressing all sites of significant atherosclerosis [2, 16]. Several studies have shown that complete revascularization can lead to lower rates of subsequent myocardial infarction, reduced need for repeat revascularization procedures, and improved overall survival compared to revascularization alone [19, 20] However, this approach is not without concerns. Complete revascularization usually requires longer procedure times and more exposure to contrast media, which can increase the risk of procedural complications such as nephropathy and contrast‐induced bleeding [21]. In addition, due to the more extensive nature of the intervention, there is concern about the possibility of increased myocardial damage and perioperative arrhythmia [22, 23]. Despite these challenges, ongoing research and clinical trials continue to investigate the optimal timing and patient selection criteria to maximize the benefits of total revascularization and minimize its risks.

Based on previous research and their identified gaps, this study aims to fill the gap in understanding the optimal strategy of total vascular repair for diabetic patients with AMI. Previous studies have shown mixed results regarding repair of the culprit artery versus complete revascularization, particularly in the setting of diabetes, which complicates both disease progression and treatment efficacy. This research, with a special focus on diabetic patients who are prone to multiple diseases and lower clinical outcomes, in a new population and in Iran, and by considering and examining new complications and more comprehensive and different clinical parameters, is vitally needed. It responds to determine whether a more comprehensive revascularization approach can improve survival and reduce complications.

## 2 Method

This was a retrospective cohort study and included diabetic patients with ST‐segment elevation myocardial infarction (STEMI) in Shahid Madani Hospital of Tabriz University of Medical Sciences (Tabriz, Iran) between April 1, 2021 and April 1, 2023. were admitted to the hospital and underwent PCI. According to the results of past studies, approximately 50% of the patients have multivessel coronary artery disease (CAD) [24, 25, 26]. Therefore, considering that the size of the population is very large, Cochran's formula will be used to determine the sample size.

$$\begin{array}{c} d=0.05\\ t=1.96\\ p=0.5\\ q=0.5 \end{array}n=\frac{t^{2}pq}{d^{2}}=\frac{{\left(1.96\right)}^{2}(0.5)(0.5)}{{\left(0.05\right)}^{2}}=\frac{0.9604}{0.0025}=384.$$

Based on this formula, we examined 384 cases in this study, which were considered as a total sample of 400 cases due to the possibility of dropping or seeing defects in the examined cases. However, based on and considering the prevalence of single vessel disease (SVD) among the target population [1], we anticipate that a proportion of patients may not meet the criteria for MVD and this phenomenon naturally It may reduce the final sample size eligible for entry.

To access the required information, methods such as data collection only based on the patient's file, or a telephone interview or a face‐to‐face visit may take place based on access to the patient. In summary, although data collection from documents is standard in retrospective studies, supplementing data through telephone calls or visits can also be valuable if access to comprehensive patient records is limited or if verification and completeness are required. Data becomes important. which were divided into two groups of 200 according to the number of the culprit arteries (one artery or multiple arteries) [26].

The researcher identified all consecutive PCI patients using interventional imaging data and assign a unique ID to each case.

Then researchers collected clinical and laboratory data using electronic medical records. The investigated items included the demographic profile of the patient, history of cardiac and noncardiac disease in the past, clinical characteristics of the patient during hospitalization, laboratory factors, complications related to the treatment method and the use of cardiac drugs during hospitalization and during discharge. Left ventricular ejection fraction (LVEF) was evaluated by echocardiography during hospitalization. Also, data related to surgical records in PCI cases was completed by the researcher.

Angiography was estimated visually or by a computer system. The degree of thrombolysis in myocardial infarction (TIMI) were determined. Finally, the clinical follow‐up in December 2023 was evaluated by visiting the hospital or telephone interview with the general physician/cardiologist of the patient, the patient or his family. All the data were from the records. Patients' medical records were obtained. If these data are not available, the required information were obtained by a telephone call to the patient's referring physician. All data were judged and classified by two cardiologists.

Patients who were eligible to enter the study must have the following criteria: (1) chest pain less than 12 h from the onset of pain to the time of catheterization, (2) significant ST‐segment elevation (at least 0.1 mV in two or limb leads or at least 0.2 mV in two or more connected precordial leads (or new left bundle branch block [LBBB]). To be included in the present study, patients must have MVD, which is defined as the presence of angiographic diameter stenosis. 50% or more is defined in at least one main epicardial coronary artery or its main branches (with diameter ≥ 2 mm). Exclusion criteria include (1) occlusion of only one coronary vessel, (2) cardiac shock, (3) any type of stented thrombus, (4) coronary artery bypass graft in the past for P‐PCI treatment, (5) the presence of nonculpable lesions in coronary artery branches with a diameter of 2 mm or less.

The studied population was divided into two groups. The first group in which only the primary lesion responsible for PCI was repaired during catheterization or hospitalization and staged total revascularization group (SR group), in which, after PCI of the main culprit lesion, PCI of the nonculprit lesion was performed within 1 month after discharge, regardless of symptoms or evidence of ischemia. The primary endpoint studied was Mortality from heart disease or nonfatal reinfarction was the secondary endpoint of death from other diseases, nonfatal reinfarction leading to any unplanned repeat PCI or vascular bypass surgery, including bleeding or nephropathy caused by contrast material, stroke, and acute or subacute thrombosis also were tracked during the hospitalization period. Stroke, as an acute event with cerebrovascular origin that causes focal neurological dysfunction for more than 24 h, will also be tracked by clinical and radiographic criteria. The volume of contrast material and creatinine were evaluated 1 day before angiography and on the first and third day. For this purpose, creatinine, urea and GFR factors in the blood test of all patients were collected and analyzed before and after angiography. The patients who had no symptoms after primary PCI and were stable during hospitalization were discharged according to their own request and were called for staged PCI within a month. We added more number of patients due to a number of patients who were discharged did not want to return due to the absence of symptoms and high functional class.

Contrast‐induced nephropathy will be evaluated as an increase in serum creatinine concentration ≥ 0.5 mg/dL or ≥ 25% above the baseline level 72 h after exposure to contrast material. Informed written consent will be formally obtained from all participants.

SPSS version 22.0.0.0 used for descriptive statistics for patient characteristics and outcomes. Comparative analysis between offender groups and total vascular staging used appropriate tests (e.g., *t*‐test, chi‐square test) for continuous and categorical variables.

## 3 Results

A total of 1186 patients were treated with P‐PCI in our center, of which 521 patients had diabetes and finally 393 patients were included in the study. Out of this number, 271 people (68.9%) only repaired the culprit vessels (group 1) and 122 people (31.1%) received complete restoration of the vessels again in stages (group 2). In the second group, the time of PCI of the nonculprit lesion was during hospitalization or within 1 month after hospitalization. The clinical characteristics of the two groups of patients were compared as shown in Table 1.

**Table 1:** Clinical characteristics of patients.

Factors	Group 1 (*n* = 271)	Group 2 (*n* = 122)	*p*‐value
Three‐vessel disease	135 (49.8%)	52 (42.6%)	*p* > 0.05
Male	178 (65.75)	79 (64.7%)	*p* > 0.05
Age	64.14 ± 10.3	61.55 ± 10.6	*p* > 0.05
Previous MI	38 (14%)	16 (13.1%)	*p* > 0.05
SMOKING	100 (36.9%)	41 (33.6%)	*p* > 0.05
Cr > 1.5	46 (16.9%)	14 (11.5%)	*p* > 0.05
Hypercholesterolemia	97 (35.8%)	40 (32.7%)	*p* > 0.05
FBS > 126	257 (94.8%)	118 (96.7%)	*p* > 0.05
Hypertension	154 (56.8%)	62 (50.8%)	*p* > 0.05
Systolic blood pressure (mmHg)	30.77 ± 136.21	141.12 ± 23.9	*p* > 0.05
Heart rate/min	81.94 ± 17.11	81.75 ± 12.76	*p* > 0.05
Symptom to balloon time, h	13.15 ± 22.18	11.94 ± 16.4	*p* > 0.05
Lateral STEMI	15 (5.53%)	7 (5.73%)	*p* > 0.05
Inferior STEMI	84 (31%)	71 (58.2%)	*p* ≤ 0.05
Anterior STEMI	170 (62.7%)	46 (37.7%)	*p* ≤ 0.05
LVEF (severely reduced)	84 (31%)	22 (18%)	*p* > 0.05
The vessel responsible—RCA	65 (24%)	49 (40.2%)	*p* ≤ 0.05
The vessel responsible—LCX	30 (11.1%)	22 (18%)	*p* > 0.05
The vessel responsible—LAD	168 (62%)	45 (36.9%)	*p* ≤ 0.05
The vessel responsible—LM	8 (2.95%)	6 (4.9%)	*p* > 0.05

Preoperative details and drugs prescribed at the time of discharge are shown in Tables 2 and 3. Patients in group 2 had more stents and longer total length of stent.

**Table 2:** Clinical information of patients before surgery.

Factors	Group 1 (*n* = 271)	Group 2 (*n* = 122)	*p*‐value
TIMI flow grade 0/1 on arrival	78 (28.8%)	46 (37.7%)	*p* > 0.05
TIMI flow grade 3 post‐PCI	262 (96.7%)	122 (100%)	*p* > 0.05
Number of stents	1.27 ± 0.72	1.37 ± 0.75	*p* > 0.05
No stenting	19 (7%)	4 (3.3%)	*p* > 0.05
Total stent length, mm	88.01 ± 43.64	99.15 ± 57.4	*p* > 0.05
Lesion site in culprit vessel			
Left anterior descending artery	16 (6%)	2 (1.6%)	*p* > 0.05
Left circumflex artery	163 (60%)	53 (43.4%)	*p* > 0.05
Right coronary artery	27 (10%)	22 (18.1%)	*p* > 0.05
Thrombus aspiration catheter used	65 (24%)	45 (36.9%)	*p* > 0.05
Creatinine before angiography	1.24 ± 0.44	1.20 ± 0.34	*p* > 0.05
Urea before angiography	20.8 ± 9.33	19.22 ± 8.17	*p* > 0.05

**Table 3:** Clinical information of patients after surgery and medications at the time of discharge.

Factors	Group 1 (*n* = 271)	Group 2 (*n* = 122)	*p*‐value
Creatinine after angiography	1.57 ± 0.86	1.23 ± 0.67	*p* ≤ 0.05
Urea after angiography	26.98 ± 16.3	21.07 ± 11.6	*p* ≤ 0.05
Aspirin	259 (95.6%)	118 (96.7%)	*p* > 0.05
Clopidogrel	108 (39.8%)	60 (49.2%)	*p* > 0.05
Ticagrelor	152 (56%)	62 (50.8%)	*p* > 0.05
Statin	201 (74.2%)	98 (80.3%)	*p* > 0.05
Beta‐blockers	89 (32.8%)	38 (31.1%)	*p* > 0.05
Angiotensin‐converting enzyme inhibitors	131 (48.3%)	69 (56.5%)	*p* > 0.05
Angiotensin receptor blockers	200 (73.8%)	84 (68.8%)	*p* > 0.05
Calcium‐channel blocker	166 (61.2%)	69 (56.5%)	*p* > 0.05
Nitrate	54 (19.9%)	36 (29.5%)	*p* > 0.05

Complications during surgery were recorded separately for each patient. All patients were followed up to an average of 12 months. During the follow‐up period, 204 (75.3%) cases of readmission, 48 (17.7%) cases of cardiac deaths, 22 (8.1%) cases of nonfatal myocardial re‐infarction, and 8 (2.9%) cases of death from other causes were observed in group 1. In the second group: 97 cases (79.5%) of readmission, eight cases (6.5%) of cardiac death, 18 cases (14.7%) of nonfatal myocardial re‐infarction were observed. The occurrence of cardiac death in the second group was significantly lower compared to the first group (*p* ≤ 0.05). Complications related to surgery can be seen in Table 4.

**Table 4:** Complications related to surgery.

Complications	Group 1 (*n* = 271)	Group 2 (*n* = 122)	*p*‐value
Major bleeding	12 (4.4%)	13 (10.6%)	*p* > 0.05
Ventricular arrythmia	31 (11.4%)	2 (1.6%)	*p* ≤ 0.05
ReMI urgent PCI	25 (9.2%)	2 (1.6%)	*p* ≤ 0.05
death	55 (20.3%)	5 (4.1%)	*p* ≤ 0.05
Other causes	12 (4.4%)	4 (3.3%)	*p* > 0.05

The present study determined the effects of different treatment strategies on diabetic patients with myocardial infarction in a real clinical setting. The main findings were as follows:

1. staged total revascularization not only significantly reduced the rate of cardiac death or nonfatal reinfarction, but also reduced the need for unplanned repeat revascularization.
2. A significant difference in cardiac mortality or nonfatal re‐infarction was observed between the two treatment groups.
3. Complete vascular repair significantly reduced intraoperative complications.

## 4 Discussion

In this study, we examined the impact of different therapeutic strategies on diabetic individuals suffering from myocardial infarction in a real‐world clinical context. The results revealed that staged total revascularization was associated with lower rates of readmission, cardiac deaths, nonfatal myocardial re‐infarction, and overall mortality when compared to CR (*p* ≤ 0.05). This benefit was primarily due to a decrease in each individual component of the composite outcome. Staged total revascularization has been shown to significantly lower the rates of cardiac death and nonfatal reinfarction, as well as to reduce the likelihood of unplanned repeat revascularization. A significant difference in the occurrence of cardiac mortality and nonfatal reinfarction was evident between the two treatment groups. Furthermore, the process of complete vascular repair was linked to a significant decrease in intraoperative complications.

The daily care of diabetic individuals who have suffered a myocardial infarction is becoming progressively more complex from therapeutic, organizational, and economic viewpoints [27, 28, 29]. The ongoing discourse highlights the resource‐intensive nature of invasive treatments and hospital stays, alongside a notable deficiency in compelling evidence from randomized trials that validate such interventions for this specific patient group. Evidence suggests that complete revascularization, whether determined through angiography or physiological evaluation, is more effective than the culprit‐only approach in younger, low‐risk patients with STEMI. This efficacy is largely attributed to a reduction in the recurrence of myocardial infarction and the need for additional revascularization [30, 31]. However, diabetic patients with myocardial infarction possess unique clinical, anatomical, and procedural characteristics that have not been adequately addressed in existing studies, including the impact of comorbid conditions, increased frailty, more complicated coronary anatomy, a higher incidence of NSTEMI, and a greater risk of complications and adverse reactions from a multidrug treatment approach. Therefore, there is an urgent requirement for targeted research to guide the management and therapeutic options for diabetic patients experiencing myocardial infarction [27, 32].

Examining the difference in the results of repair of the culprit vessels compared to complete repair in diabetic patients with AMI is significant for several reasons. Considering the complex interplay between diabetes and cardiovascular disease, especially the increased risk of adverse outcomes in diabetic patients, it is very important to optimize treatment strategies. Diabetic patients often present with multifactorial CAD and are more likely to experience complications such as heart failure, reinfarction, and death after AMI. This warrants careful consideration of whether total repair, which addresses all significant stenosis, provides better outcomes compared to a traditional approach that treats only the culprit artery.

Recent studies highlight the potential benefits of complete revascularization. For example, in a clinical trial in 2023, Zardasht Oqab et al. showed that total revascularization significantly reduced major cardiovascular events compared with PCI of the culprit vessel alone in STEMI patients with diabetes [2] and also Ibrahim C Kaya et al showed in a study in 2023 that complete vascular repair in NSTE‐ACS and unstable angina patients, including diabetic patients, is possible with low mortality and complication rates and promising survival results [19] thus recent studies showed in total revascularization significantly reduces major cardiovascular events compared with PCI of the culprit vessel alone in STEMI patients with diabetes. And similarly, promising survival and low complication rates with complete repair have been shown in NSTE‐ACS and unstable angina patients, including diabetic patients. These findings suggest that a more comprehensive approach to complete repair may improve long‐term outcomes by reducing overall ischemic burden and preventing future cardiovascular events. However, the increased complexity of the procedure and the risks associated with complete reconstruction, such as longer operative times and exposure to contrast media, necessitate further research to identify patient selection criteria and optimal timing for this approach.

Based on previous research and their identified gaps, this study aims to fill the gap in understanding the optimal strategy of total vascular repair for diabetic patients with AMI. Previous studies have [2, 19] shown mixed results regarding repair of the culprit artery versus complete revascularization, particularly in the setting of diabetes, which complicates both disease progression and treatment efficacy. This study, with a special focus on diabetic patients who are prone to multiple diseases and poorer clinical outcomes, in a new population and in Iran, and by considering and examining new complications and more comprehensive and different clinical parameters, is vitally needed. It responds to determine whether a more comprehensive revascularization approach can improve survival and reduce complications.

The study's cohort had a median age of 63.4 years, which is comparable to the median ages reported in previous key trials within this area of research. Patients with diabetes are known to have a heightened incidence of concurrent health issues, including peripheral artery disease and chronic kidney disease, leading to a significantly higher rate of adverse events than those documented in earlier studies. The rise in adverse events was predominantly linked to instances of death and myocardial infarction. Typically, older patients are less likely to undergo elective invasive coronary procedures compared to younger individuals. However, the findings from our trial indicated that the risk reduction associated with staged complete revascularization (SR group) among diabetic patients was consistent with prior research outcomes. Moreover, the benefits of complete revascularization were observed to increase over time, as indicated by the persistent divergence of the Kaplan–Meier curves during the initial year.

In contrast to prior investigations, our study focused solely on patients suffering from STEMI. The safety of staged complete revascularization in myocardial infarction cases depends significantly on the accurate differentiation between the culprit lesion and nonculprit lesions. Our research demonstrated that staged complete revascularization is both feasible and safe for STEMI patients, contingent upon the clear identification of the culprit lesion via electrocardiography, echocardiography, and angiography, as required by the trial protocol.

The rationale for utilizing coronary physiology in diabetic patients is primarily to reduce the number of interventions by selectively treating only those nonculprit vessels that are prognostically significant during the intervention for the culprit vessel. This strategy aims to mitigate the risk of complications that could adversely affect prognosis. The potential benefits are not confined to periprocedural complications, such as stroke, contrast‐induced acute kidney injury, and periprocedural myocardial infarction. The extent of dual‐antiplatelet therapy is significantly influenced by the number of vessels treated and stents placed, which is associated with an elevated risk of major bleeding and mortality in patients vulnerable to such complications.

This trial has inherent limitations that must be acknowledged. The open‐label nature of the study may have led to biases among both patients and physicians concerning subsequent revascularization decisions in the culprit‐only treatment group, as they were privy to the angiographic results. Notably, events necessitating ischemia‐driven revascularization accounted for a relatively small segment of the total primary outcome events, whereas more severe clinical outcomes, including myocardial infarction and death, dominated the event count. Since complete revascularization was guided by coronary physiological assessments, one should exercise caution when considering the applicability of these results to angiography‐guided complete revascularization, particularly given the unique attributes of the trial population. Additionally, the revascularization was conducted during the index hospitalization with the use of sirolimus‐eluting, biodegradable‐polymer, ultrathin stents, which raises questions about the relevance of our findings to patients treated with different management approaches and stent types.

## 5 Conclusions

In patients with diabetes, the implementation of staged complete revascularization resulted in a reduced incidence of the composite outcomes, which include readmission, cardiac mortality, nonfatal myocardial reinfarction, and total mortality, compared to revascularization focused solely on the culprit lesion.

## Author Contributions

N.A., S.M., M.R., and A.A. contributed to the data gathering, conceptualization, supervision, and writing the original draft. M.R. and A.H. reviewed and edited the final manuscript. All authors read and approved the final manuscript.

## Conflicts of Interest

The authors declare no conflicts of interest.

## Data Availability

The data that support the findings of this study are available on request from the corresponding author. The data are not publicly available due to privacy or ethical restrictions. Supplementary material is available in the online version.
